# Media-multitasking and cognitive control across the lifespan

**DOI:** 10.1038/s41598-022-07777-1

**Published:** 2022-03-14

**Authors:** Natasha Matthews, J. B. Mattingley, P. E. Dux

**Affiliations:** 1 School of Psychology, The University of Queensland, St Lucia, QLD 4066 Australia; 2 Queensland Brain Institute, The University of Queensland, St Lucia, QLD 4066 Australia; 3 Canadian Institute for Advanced Research (CIFAR), Toronto, Canada

**Keywords:** Cognitive neuroscience, Human behaviour, Psychology

## Abstract

The exponential rise in technology use over the past decade, and particularly during the COIVD-19 pandemic, has been accompanied by growing concern regarding the consequences of this technology use for our cognition. Previous studies on the influence of technology-multitasking (the use of two or more technologies simultaneously) on cognitive performance have provided mixed results. However, these past studies have generally ignored the considerable developmental trajectories that cognitive abilities undergo across the lifespan. In a large community-based science project we investigated the relationship between media-multitasking and cognitive flexibility (multitasking ability) in participants aged 7–70 years. Higher levels of every-day technology multitasking were associated with higher levels of multitasking performance across an age range in which multitasking ability undergoes developmental change. These findings suggest that age is an important moderator of the relationship between technology use and cognition.

## Introduction

The last few decades have seen the introduction of a wide variety of devices and technologies for consuming and sharing information. For many people, young and old, devices such as smartphones and tablets, as well as media platforms such as Facebook, Twitter and Instagram, have become a ubiquitous part of life. Indeed, it is estimated that more than 50% of US consumers use a smart phone^1^. Rates of smartphone usage are particularly high among children and teens^2^, but even individuals over the age of 65 years are significant users of such technology^3^. The portability and instant availability of multimedia technology encourages behavior known as “media-multitasking”, i.e., monitoring and interacting with multiple streams of information simultaneously while also engaging in other tasks. Among adolescents, media-multitasking has been increasing rapidly. It is estimated that on average American youths aged 8–18 years spend approximately 10 h a day using technology. Of this time, 29% is spent engaged in media-multitasking, e.g., playing online games while instant messaging. This figure is up from 16% just a decade ago^4^ and has seen a dramatic increase during the COVID-19 pandemic with so much of the world’s population under stay at home orders^5^. Here, in a large sample, we ask whether engaging in media-multitasking across the lifespan is associated with changes in actual multitasking ability, as assessed by objective measures of cognitive performance.

A long history of work in psychology has shown that multitasking, which relies on high-level cognitive control operations^6^, leads to costs in behavioral performance^7,8^. For this reason, there has been significant interest within the scientific community and the wider general public concerning the impact on cognition of chronic multimedia exposure^9–13^, particularly in children whose brains are still in a critical stage of development^14^. A major topic of investigation on the long-term influence of everyday media-multitasking on basic psychological operations has focused on how media-multitasking relates to processes associated with avoiding distraction in young^9,11,13,15^. In addition, in an important and extensive review, Uncapher and Wagner^16^ present a wide range of other research examining media-multitasking and its influence across different domains of cognition such as those involved in impulsivity, memory and vigilance. For example, Ophir and colleagues^13^ found that “high” media-multitaskers are more susceptible to irrelevant or distracting stimuli than individuals who engage in low levels of media-multitasking. Such findings suggest that high media-multitasking is associated with a reduced capacity to focus attention. However, findings from similar studies have been inconsistent^9,13^, possibly due to reliance on small participant samples^15^ and a failure to account for the full continuum of media-multitasking behavior by employing analyses based on groups at the extremes of distributions rather than using regression approaches to examine the whole sample^17^. Indeed, Uncapher and Wagner^16^ highlight that answering key outstanding questions regarding media-multitasking and cognition will require larger-scale samples that draw from individuals of all ages, media use histories, demographics, and neurocognitive profiles.

Here we explore how multimedia exposure influences an individual’s cognitive control ability. Of import, we did this using the largest sample size to date via a community-based project where we tested 1511 individuals. In addition, we focussed on two untested aspects relating to media-multitasking: how this influences the ability to managing multiple cognitive tasks concurrently generally and how this changes across the lifespan (we tested ages ranging from 7 to 70 years). Multitasking ability is associated with substantial maturational change from childhood through to adolescence^18^. During this age period there is also the potential for considerable neural plasticity in brain regions involved in cognitive control^19^. However, currently, it is not known how these developmental changes moderate the relationship between pervasive technology use and cognitive control^18,20^. High levels of technology multitasking have been associated with both impaired executive function^21^, and improved inhibitory control in early adolescents^22^. At the same time, multitasking ability is known to decline with age^23^. Thus, it remains unknown whether such changes across the life-span are influenced—for better or worse—by multimedia technology use. Indeed, in a recent review, Beuckels et al.^24^ identified that there is an important research gap in the field of media multitasking, in which younger and older media multitaskers have received little to no research attention. Thus, our research addresses an important outstanding issue in the field.

Testing was conducted via a purpose-built interactive museum xhibit. Participant performance on the exhibit was supervised to ensure data quality and reliability. Participants undertook a multitasking test on a digital tablet. This test comprised of three visual tasks, each of which was presented under dual-task and single-task conditions. Of primary interest were participants’ multitasking costs measured by subtracting dual-task from single-task performance measures. Participants also completed a measure of everyday media-multitasking based on instruments used by Pea et al.^25^. From this multitasking survey, a media-multitasking index (MMI) was calculated for each participant, which reflected the average number of media used by an individual at any given time^25^.

## Method

### Participants

**Figure 1:**
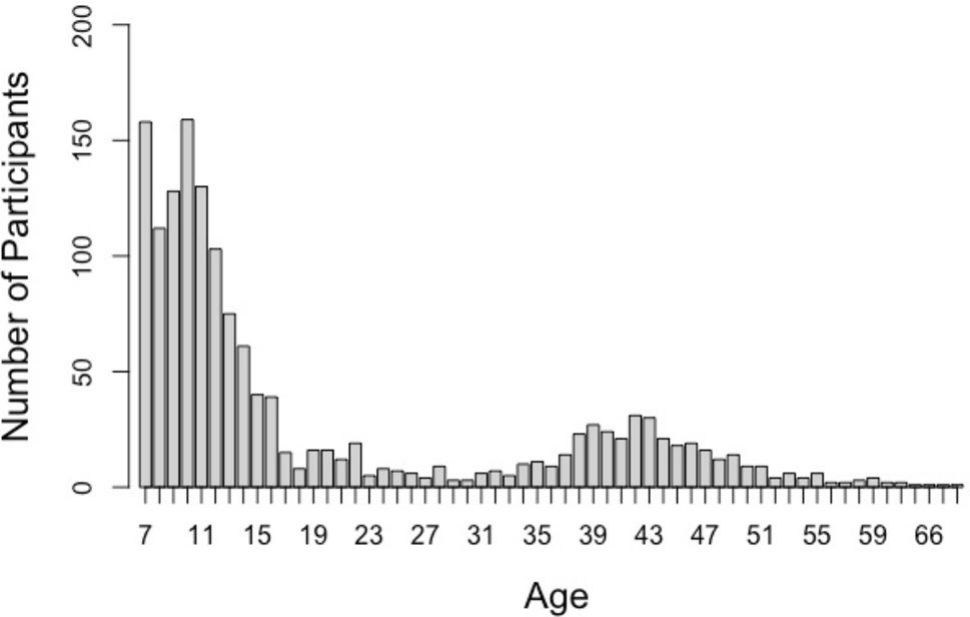
Frequency distribution of study participants by age.

Members of the community were recruited from among visitors to Questacon, The National Science and Technology Centre located in Canberra, Australia. All visitors to the Center over a 2-week school-holiday period were invited to participate in the study, which was set-up as an interactive, supervised exhibit. The exhibit, provided visitors with an opportunity to explore their multitasking ability, and to learn about multitasking costs. Prior to commencing the study, participants read an information statement and provided informed consent to their data being used for research purposes (informed consent was obtained from a parent and/or legal guardian for children and adolescents). The study protocol was approved by The University of Queensland’s Human Research Ethics Committee and all methods were carried out in accordance with relevant guidelines and regulations. Over the course of the study period, 1855 individuals initiated an interaction with the exhibit. Of these individuals 1511 met criteria for inclusion. Data from individuals were excluded from analysis if they did not provide informed consent (*n* = 47), if they were less than 7 years or greater than 70 years of age (*n* = 24), or if they failed to provide valid data for each of the study components to allow for matching across datasets (*n* = 273). In the interest of retaining as diverse a sample as possible, valid data for each study component was defined to indicate that participants attempted to engage with that portion of the study. This was defined for the demographics survey as a response to all questions, for the media-multitasking survey as response to at least one of the media categories, and for the multitasking test as a response recorded for at least one task item. The mean age of participants was 19.56 years (*SD* = 14.56 years; Fig. 1), and 56% of the sample were female. The shape of the age distribution likely reflects the fact that most visitors to Questacon are children under 12 years of age, accompanied by their parents who were aged in their early to mid 40s. As this was a convenience sample, collected as part of an ongoing exhibit we placed no restrictions on the number of participants who completed the study and aimed to collect data from as many participants as possible in the time–frame provided.

### Procedure

Study data were collected as part of a supervised exhibit. The exhibit area included six participant workstations and was shielded on three sides from the surrounding museum environment by partitions. Two research assistants were present at all times to coordinate the flow of participants through the exhibit and to oversee task performance. The design of the study protocol was motivated by the need to provide a robust measurement of multitasking ability that could be performed by museum visitors across a large age range, and within a short timeframe so as to ensure participant engagement.

**Figure 2:**
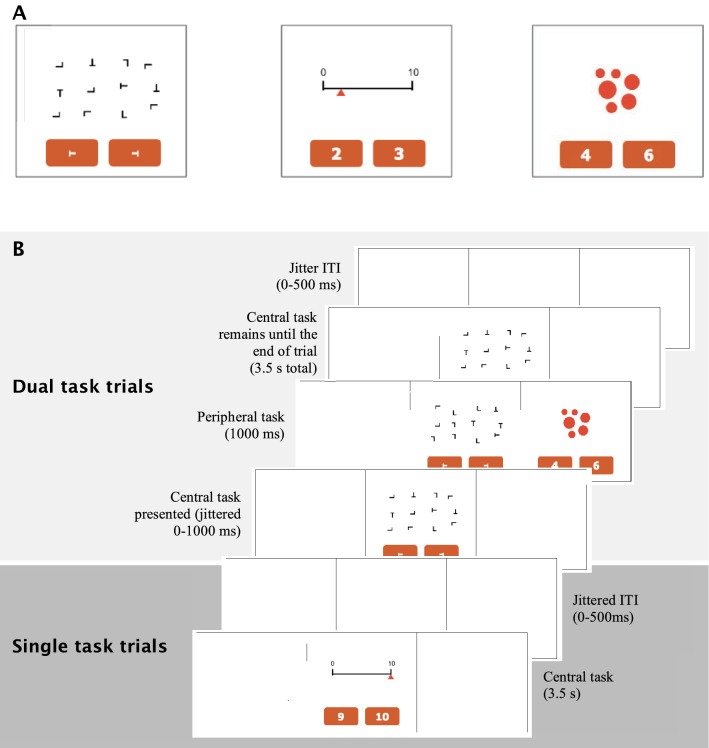
Schematic of the multitasking test used in the study. (**A**) Example displays from the three tasks included in the multitasking test; visual search (left), number-line estimation (middle), and dot enumeration (right). Participants responded to the three visual tasks by pressing one of the two answer alternatives shown in orange using the touch screen function of a digital tablet. (**B**) Timing of displays in the multitasking test. Participants were presented with randomly interleaved single-task (lower rows) and dual-task trials (upper rows). The task display consisted of three panels (centre, left and right). During *single-task trials* one task was presented alone in the central panel (either visual search or number-line estimation; 3, 500 ms), or alone in a peripheral panel (dot enumeration task; 1000 ms). During *dual-task trials* the central task (number-line estimation or visual search) was presented in the central panel, and after a jittered inter-stimulus-interval (ISI) of 0–1000 ms, the dot enumeration task was presented in either the left or right peripheral panel for 1000 ms. The inter-trial interval was jittered between 0 and 500 ms. Participants were instructed to respond as quickly and as accurately as possible. On dual-task trials participants were free to choose to perform either of the tasks displayed, and were free to switch between them at will if desired.

The study had three components, all of which were presented on a digital tablet (Apple iPad, V4), and total took approximately 15 min to complete. These components included: (1) a short demographic questionnaire; (2) a technology-multitasking survey, in which participants estimated the amount of time they spent each day engaged in using a variety of digital platforms and devices and (3) a multitasking test in which participant performed three cognitive tasks under single- and dual-task conditions (Fig. 2). Instructions were provided on the screen prior to each component of the study, and a trained experimenter was present to assist where further explanation was necessary. Practice trials, which participants were free to undertake until they understood and felt comfortable with the tasks, were provided prior to the multitasking test. All components of the study were run through a website built in the web application language PHP, and employed a MySQL database platform for data storage. Presentation and scoring of the tests was done using JavaScript, which gave a timing accuracy within 15 ms. The visual stimuli were made using simple graphics and HTML colours.

### Media-multitasking survey

Our media-multitasking survey was based on the work of Pea et al.^25^, whose media-multitasking survey provides a robust assessment of categories of technology inclusive of those used by children and adolescents. The survey assessed the extent to which individuals used different forms of technology simultaneously in their everyday lives. Specifically, participants were asked to estimate the number of hours in an average day they spent: (1) reading, studying or doing homework; (2) watching video content, including YouTube, TV or movies, (3) playing video games, (4) listening to music, (5) using social media (including email, Facebook, Instagram, Snapchat, etc.), (6) texting or instant messaging, and (7) talking on the phone or video chatting. As in Pea et al.^25^, for each category participants rated their use on a multiple-point scale with the following options: never, less than 1 h, about 1–2 h, about 2–3 h, about 3–4 h, or more than 4 h. For each medium for which participants indicated a response other than “never”, they were also presented with a follow-up question asking them to indicate the amount of time (rated on the same multiple-point scale for hours of use) they spent concurrently engaged with the other media types.

The key measure from this survey was the media-multitasking index (MMI; Pea et al.^25^). To generate this value for each participant, for each primary medium we summed the *proportion* of time participants also engaged in using other media simultaneously, and then multiplied this value by the total time the individual spent using the primary medium. These values, for the seven primary media, were then summed for each participant, yielding a final MMI^25^.

### Multitasking test

The multitasking test comprised of three visual tasks displayed on a digital tablet (Fig. 2A), each of which were presented under dual-task and single-task conditions. These tasks were chosen to allow for a time-efficient, yet robust estimate of multitasking as they each tapped various aspects associated with numerical cognition and visual attention. All of these tasks have been employed previously in a range of multitasking studies and thought to tap overlapping substrates in the parietal lobe, e.g.,^25^. Tasks with overlapping neural substrates have been demonstrated to produce greater multitasking costs^27^. The multitasking test took approximately 5–10 min to complete and were simple enough to be undertaken by both young children and elderly individuals, but were designed to avoid ceiling effects in adolescents and adults. Individual tasks were presented in one of three panels across the screen (left, center, right; Fig. 2B). All tasks had two response ‘buttons’ located beneath the stimulus material within each panel, and participants made their response by tapping one of these buttons as quickly as possible. The background of the panels was white, the stimuli were black and red, and the response buttons were orange.

The *center panel* contained either a visual search task^28^ or a number-line estimation task^29^. A dot enumeration task^30^ was presented concurrently with the center task, or following a variable temporal offset (see below), in the left or right *peripheral panel*. Participants were instructed to respond as quickly and accurately as possible to all tasks. Trials in which one of the three tasks was presented alone (*single-task condition*) and those in which both a central and peripheral task appeared concurrently (*dual-task condition*) were randomly intermixed (Fig. 2B). On dual-task trials, participants were free to choose which task to perform first, and were not restricted in how often they could switch between tasks. This arrangement elicited a classic multitasking scenario, in which participants had to monitor multiple sources of information concurrently (each of which drew on a distinct set of cognitive processes), and select which task to perform or ignore at any given moment in time^7^.

The key dependent measure was multitasking cost, which was calculated by subtracting scores under single-task conditions from those under dual-task conditions, separately for accuracy and reaction time for each of the three tasks. As one aim of the exhibit was to educate museum visitors about multitasking and multitasking costs, at the completion of the task, participants were presented with a bar chart of their performance on the dot enumeration task (our key multitasking measure) under single- and dual-task conditions. They were also provided with a short, written passage on multitasking and an explanation of what multitasking costs represent. For the analysis of the relationship between MMI and multitasking ability we focussed on multitasking costs for the dot enumeration task as our measure of multitasking ability as it was the peripheral task (i.e., appeared in the periphery of the screen) and consequently designed to show the largest multitask costs. Indeed, extensive research on multitasking effects has shown that generally primary tasks are not influenced by multitasking manipulations to the same extent as peripheral/secondary tasks (see Pashler^8^ for a review).

#### Central tasks

Two of the tasks (visual search and number-line estimation) were always presented in the center panel. Visual search: search array stimuli were presented in an invisible 4 × 4 grid, with item position jittered. On each trial, 12 stimuli were presented; 11 of these were distractors (upright Ts, upside down Ts, upright Ls, upside down Ls or Ls rotated 90° to the left or right) and one was the target—a T rotated 90° left or right. The positions of all stimuli were pseudo-randomly determined on each trial. Participants indicated whether the target T was oriented to the left or right by tapping the corresponding response button beneath the search display (Fig. 2A left). Which of the two positions (left or right on the screen) corresponded to the correct answer was randomized on each trial. Stimuli were presented for 3500 ms, followed by an ISI jittered between 0 and 500 ms. Number-line estimation: participants were presented with a number line with tick labels at “0” and “10” for reference (Fig. 2A center**)**. On each trial a red triangle was situated below the line and participants were asked to estimate an integer that corresponded with the position to which the triangle pointed. Two response options were provided: one option corresponded to the correct answer, and the other was within +/− three integer values of the correct answer (e.g., in Fig. 2A, the triangle is pointing to the position corresponding to “2”, and the response options include that answer as well as a second option, in this case “3”). Which of the two response option positions (left or right on the screen) contained the correct response was randomized across trials. Stimuli were presented for 3500 ms, followed by an ISI jittered between 0 and 500 ms.

#### Peripheral task

The third task was always presented in one of the two peripheral locations (left or right panels, with equal likelihood). The side of presentation was pseudo-randomly determined on each trial. Dot enumeration task: on each trial, an array of filled red dots was presented for 1000 ms. The size of each dot was varied across trials. Participants were asked to determine whether the briefly presented dot cloud contained 4 or 6 dots (Fig. 2A right). Participants made their response by touching one of two response buttons (“4” or “6”) beneath the display. Which of the two response option positions (left or right on the screen) contained the correct response was randomized on each trial. After the dot cloud disappeared the response options persisted on the screen until the end of the trial (3500 ms) to align with the central task trials. The ISI was jittered between 0 and 1000 ms.

#### Single and dual-task trials

Each of the three tasks was presented under single- and dual-task conditions (Fig. 2B). Single task: there were 36 central task single trials (18 number line, and 18 visual search) in which only the central task was presented on the screen (e.g., lower panel Fig. 2B). There were also 36 peripheral task single trials (18 presented on the left and 18 presented on the right) in which only the dot enumeration task was presented on the screen. Dual-task: there were 36 dual-task trials in which both a central task (visual search or number line) and the dot enumeration task (on the left or right) were presented (e.g., upper panel Fig. 2B). The two tasks were either presented simultaneously or the dot enumeration task followed the presentation of the central task after a brief delay of between 0 and 1000 ms. There were an equal number of pairings of central task type with peripheral task location (9 trials in each cell).

## Results

### Overall multitasking performance

**Figure 3:**
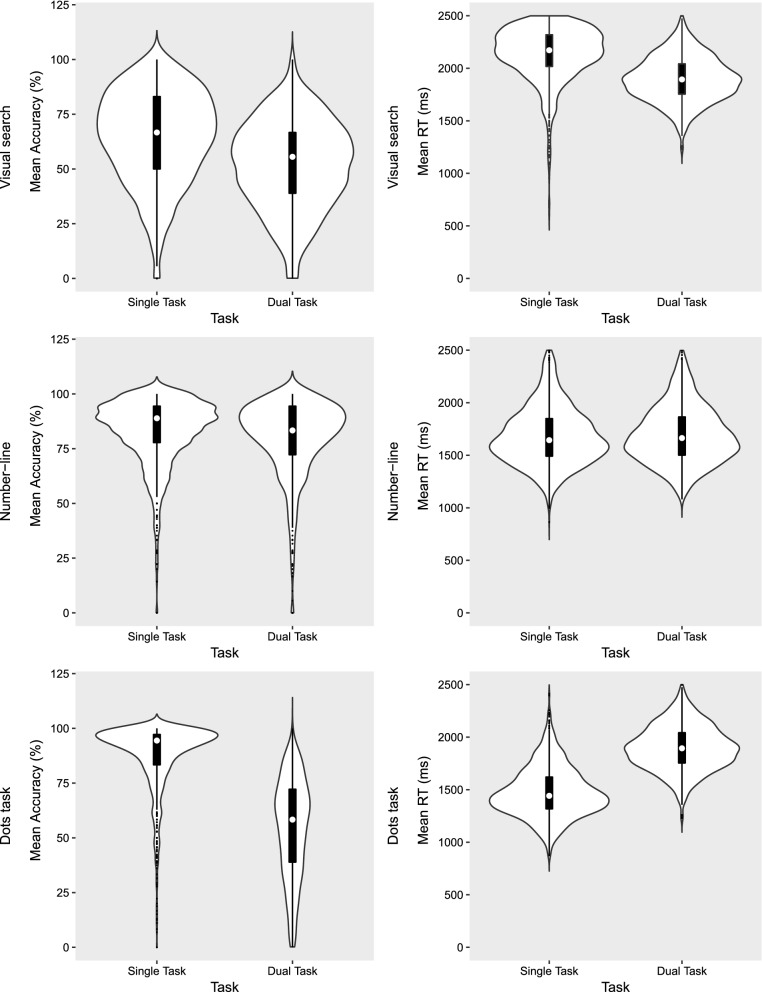
Violin plots of distributions for accuracy (percent correct; left column) and reaction time (in milliseconds; right column). (**A**) Visual search task. (**B**) Number-line estimation task. (**C**) Dot enumeration task. The white circle in each plot represents the median, the black band represents the first quartile (lower) to third quartile (upper), and the highlighted white area represents the density trace of values.

Multitasking costs, for both RT and accuracy, were calculated for each of the three tasks used in the multitasking test (Fig. 3). Visual search task: there was a significant multitasking cost for the visual search task. Participants performed significantly more accurately on single- (*M* = 64.51, *SD* = 21.00) compared with dual-task trials (*M* = 51.66, *SD* = 20.2, *F*(1, 1507 = 729.32, *p* < 0.001, η_p_^2^ = 0.326). There was evidence of a speed accuracy trade-off, such that RT was significantly faster on dual-task trials (*M* = 2186.17, *SD* = 314.04) than on single-task trials (*M* = 2203.92, *SD* = 302.53, *F*(1, 1481 = 5.026, *p* < 0.025, η_p_^2^ = 0.003) (Fig. 3A). Number-line estimation: there were significant multitasking costs for both accuracy and RT in the number-line task. Participants performed significantly more accurately on single- (*M* = 82.72, *SD* = 15.38) than on dual-task trials (*M* = 80.08, *SD* = 17.19, *F*(1, 1509 = 58.51, *p* < 0.001, η_p_^2^ = 0.037), and also responded more quickly on single- (*M* = 1690.22, *SD* = 290.04) versus dual-task trials (*M* = 1702.84, *SD* = 290.25, *F*(1, 1498 = 9.43, *p* = 0.002, η_p_^2^ = 0.006; Fig. 3B). Dot enumeration: there was a significant multitasking cost for *accuracy* on the dot enumeration task. Participants performed significantly more accurately on single- (*M* = 87.79, *SE* = 0.39) compared with dual-task trials (*M* = 45.76, *SE* = 0.59, *F*(1, 1503 = 5711.99, *p* < 0.001, η_p_^2^ = 0.777). Accuracy was higher when the dot enumeration task was presented in the left panel (*M* = 68.22, *SE* = 0.46) than in the right panel (*M* = 65.33, *SE* = 0.44, *F*(1, 1503 = 71.77, *p* < 0.001, η_p_^2^ = 0.044). There was also a significant presentation side by task interaction *F*(1, 1509 = 93.52, *p* < 0.001, η_p_^2^ = 0.058). The effect of presentation side was significant under dual-task conditions (*t*(1510) = 10.83, *p* < 0.001), but not under single-task conditions (*t*(1509) = − 1.25, *p* = 0.21). There was also a significant multitasking cost for *reaction time*, such that participants responded more quickly on single- (*M* = 1460.05, *SE* = 5.93) than on dual-task trials (*M* = 1915.02, *SE* = 0.5.78, *F*(1, 1449 = 10,724.88, *p* < 0.001, η_p_^2^ = 0.881). There was also a significant effect of presentation side, with faster reaction times when the dots were presented in the left panel (*M* = 1683.01, *SE* = 0.5.64) than in the right panel (*M* = 1692.05, *SE* = 5.74, *F*(1, 1449 = 7.19, *p* = 0.007, η_p_^2^ = 0.005), and a significant presentation side by task interaction *F*(1, 1449 = 30.78, *p* < 0.001, η_p_^2^ = 0.021). The effect of presentation side was present for both dual-task (*t*(1451) = 4.585, *p* < 0.001, and single-task trials (*t*(1449) = − 2.71, *p* = 0.007, but the effect was larger for the dual-task trials (*p* < 0.001; Fig. 3C). Thus, as was intended with the design of the study, the dot enumeration task showed the largest and most consistent (across both accuracy and RT) multitasking costs as would be expected of a peripheral/secondary task^8^.

The analysis of the relationship between MMI and multitasking ability focussed on multitasking costs for the dot enumeration task as this was our key measure of multitasking ability (see “Methods” section above). We explored the relationship between indices of multitasking and age in two ways. First, we investigated the bivariate relationship between both MMI and multitasking costs with age. We then investigated whether age moderates the relationship between MMI and multitasking costs.

### Bivariate relationships between MMI and Multitasking costs with age

**Figure 4:**
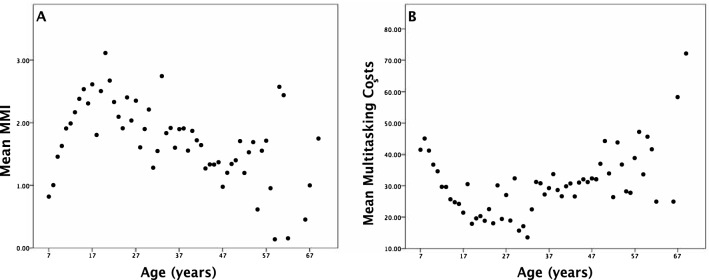
The relationship between age and indices of multitasking. (**A**) Mean MMI scores as a function of participant age. (**B**) Mean multitask cost as a function of participant age.

Both MMI (Fig. 4A) and multitasking costs for the dot enumeration task (Fig. 4B) demonstrated a U-shaped relationship with age that was best captured by a quadratic function. Mean MMI was lowest in young children and older adults, peaking for those in their mid- to late-20s (Δ*F*(1, 1507) = 90.674, *p* ≤ 0.001, *R*^2^ change = 0.057). A quadratic function fit to the data indicated that peak MMI scores (representing greatest everyday multitasking) were observed for individuals 28.1 years old. Average multitasking costs followed an inverse pattern to that of MMI and age, such that multitasking costs decreased from early childhood and adolescence, before increasing again after the age of 30 (Δ*F*(1, 1507) = 90.674, *p* ≤ 0.001, *R*^*2*^ change = 0.057). A quadratic function fit to the data indicated that the lowest multitasking costs (indicating superior multitasking ability) were found for individuals aged 34.5 years. Thus, media-multitasking and multitasking ability show substantial change with age, both peaking for individuals in their late 20 s to the early 30 s.

### Effect of age on the relationship between MMI and multitasking costs

**Figure 5:**
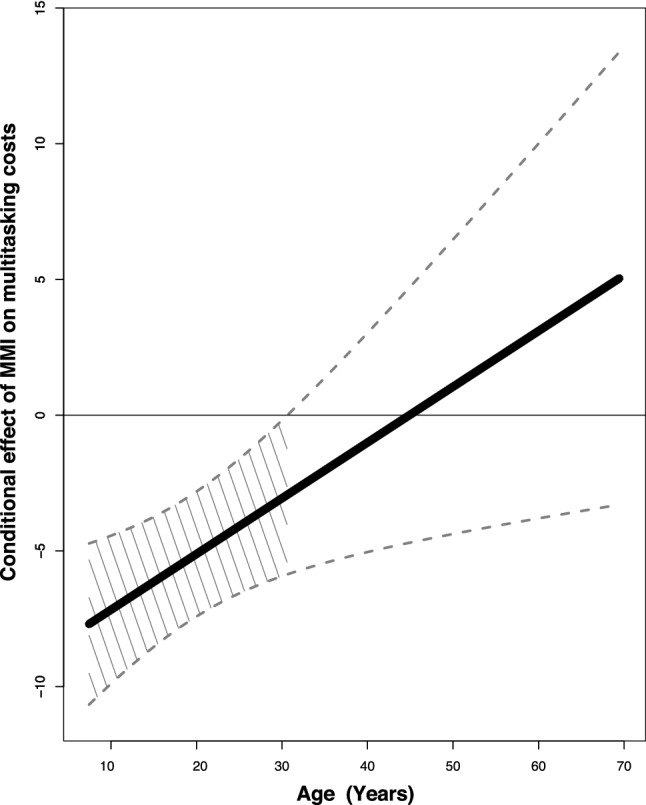
Johnson–Neyman interaction plot for the moderating effect of age on the relationship between MMI and multitasking cost. The Y-axis depicts values for the adjusted effect of MMI on multitasking cost. The bold line represents values of the adjusted effect that correspond to the full range of ages. The dashed lines show the 95% confidence interval around the adjusted effect of MMI on multitasking costs. The shaded area is the Johnson–Neyman significance region, i.e., the period over which the relationship between age and the conditional effect of MMI on multitasking cost is significant.

To determine how change across the life span moderates the relationship between everyday media-multitasking and multitasking ability we next performed a linear regression analysis with MMI as the predictor variable and age as the moderator. Variables were centred to eliminate multicollinearity. This model accounted for a significant portion of the variance in multitasking osts, *F*(3, 1506) = 16.87, *p* < 0.001, *R*^2^ = 0.034. Critically, while both age (*β* = − 0.16, *t*(1506) =  − 5.01, *p* < 0.001) and MMI (*β* = − 1.26, *t*(1506) =  − 4.24, *p* < 0.001 were significant predictors of multitasking costs, and there was also a significant interaction between these two factors (*β* = 0.05, *t*(1506) = 2.12, *p* = 0.03), indicating that the relationship between MMI and multitasking costs varies with age. This result also held when the standardized residuals of the relationship between single-task and dual-task performance on the dot enumeration task was used as the measure of multitasking costs in the regression analysis, a step that controls for the influence of single-task performance (As the multitasking cost measure is a subtraction, it is possible that variation in either single-task, or dual-task performance may have driven the observed effect. To check for this possibility, the regression analysis was re-run with the standardized residuals of the relationship between single-task and dual-task performance on the dot enumeration task as the measure of multitasking costs. This controls for the influence of single-task performance. The results were consistent with those reported above, a linear regression model with MMI as the predictor variable and age as the moderator significantly predicted variance in multitasking residuals (*F*(3, 1506) = 31.39, *p* < 0.001, R^2^ = 0.06. While both age (β = 0.02, *t*(1506) = 7.28, *p* < 0.001), and MMI (β = 0.01, *t*(1506) = 5.26, *p* < 0.001 were significant predictors of multitasking residuals, there was also a significant interaction between them (β = -0.003, *t*(1506) = -2.21, *p* = 0.03)). The Johnson–Neyman technique was employed to further explore this interaction by finding the age range over which the relationship between MMI and multitasking ability was significant (*p* < 0.05). As shown in Fig. 5, there was a positive relationship between MMI and multitasking ability for younger participants. This suggests that increased MMI (i.e., more everyday technology multitasking) is associated with reduced multitasking costs (i.e., superior multitasking performance). This relationship was significant from the youngest age group in our cohort (7 years) through to 29.25 years (*t*(1506) = − 1.916, *p* = 0.05, *β* = − 0.7609; shaded region in Fig. 5). When combined with the bivariate results showing that multitasking ability reaches its peak in the early 30s in our cohort, this result suggests that multitasking ability is positively related to multi-media use exclusively for ages over which the former ability is developing. It should be noted, that we did not have an even sampling of participants across the age range investigated. Specifically, for the age range (7–29 years) across which we observe a positive relationship between media-multitasking and multitasking performance, we had *n* = 1130 participants, however above this age range we still had *n* = 381 in older ages. Thus, given the large sample in this older age window it is unlikely that smaller participant numbers led to spurious results.

While multitasking costs on the peripherally presented dot enumeration task were our a priori focus, for completeness we also present here the regression model repeated with multitasking costs on the two central tasks (visual search task and number-line task) as the outcome variables. When performance on the number-line task was used as the outcome variable, the model accounted for a significant portion of the variance in multitasking osts, *F*(3, 1509) = 6.54, *p* < 0.001, *R*^2^ = 0.013. While both age (*β* = − 0.08, *t*(1506) = -3.50, *p* < 0.001) and MMI (*β* = − 2.32, *t*(1506) = − 2.60, *p* = 0.009 were significant predictors of multitasking costs, in this instance there was no significant interaction between these two factors (*β* = − 0.009, *t*(1506) = 0.14, *p* = 0.88). For the visual search task the overall model was not significant *F*(3, 1507) = 0.917, *p* = 0.43. As shown in Fig. 3, multitasking costs for these central tasks were smaller and more inconsistent than that seen in the dot enumeration task, and supports the selection of the dot (peripheral) task as our primary measure.

## Discussion

Collectively, the findings suggest that higher levels of media-multitasking are associated with better multitasking performance (as assessed in cognitive tests), but only for individuals aged ~ 7 to 29 years. This new observation might explain some of the variability in previous findings on media-multitasking, which were based exclusively on young adult populations (e.g., university undergraduates). These individuals likely straddle the peak developmental age for cognitive control (with the exact age determined by the developmental trajectory of the behavioral assays employed). Interestingly, in our data the sign of the relationship between multitasking costs and multi-media use also changes with age from positive in young participants to negative in older participants, suggesting that the demographic composition of participant groups may have significantly influenced the pattern of results observed in previous studies. Indeed, in a recent review, Beuckels et al.^24^ highlight that older adults are extensive media-multitaskers and note that this group have received little to no research attention, thus the present work addresses an important gap in the literature.

While it is possible that the high volume of everyday media-multitasking in children and young adults has the effect of “training” the brain to be better at handling multitasking^31^, the association observed here might instead reflect parallel trajectories in media use and cognitive development. At the same time that multitasking abilities are being established, children devote more of their newfound skills to the various digital technologies at their disposal. In addition, while media-multitasking in children and young adults was associated with cognitive benefits in our study, there might also be a range of potential negative consequences. It has been proposed that technology use steals time from other activities that could have a benefit for cognitive and social development^18^. For example, many social skills are known to develop during childhood and young adulthood and it has recently been suggested that the social isolation created through excessive technology multitasking is associated with negative social outcomes in this age group^25^.

The parameter space over which increased consumption of media, particularly through the use of new technologies, can influence cognition is vast. While the present results do not provide a definitive picture of the extent to which, and potentially how, *all* cognitive operations are influenced by media multitasking, the present results do reveal that exposure to media-multitasking is associated with changes in cognitive control. Importantly, and for the first time, we also show that this relationship varies across the lifespan and that there is a particular influence at earlier stages of life. Future large-scale studies will need to build on this work with greater numbers of measures included to measure a wider range of cognitive constructs. These will be crucial if we are to gain a better understanding of how media consumption influences development of the key cognitive operations involved in everyday life. This of course is of particular importance in the current climate as the COVID-19 pandemic has led more people to use more digital devices more frequently and across more context as a crucial part of their everyday work at home. It is likely such increased exposure has important impacts upon cognition and should be the focus of future extensions of this work.

## Data Availability

Data underlying analyses reported in this paper are available through The University of Queensland’s eSpace data repository.
